# Prävention und Management von COVID-19-Ausbrüchen auf Handelsschiffen: Technische, organisatorische und persönliche Schutzmaßnahmen zur Risikominimierung in einem besonderen Arbeitsumfeld

**DOI:** 10.1007/s40664-021-00440-y

**Published:** 2021-08-25

**Authors:** Dorothee Dengler, Thomas von Münster, Ann-Christin Kordsmeyer, Lukas Belz, Natascha Mojtahedzadeh, Jan Heidrich, Elisabeth Hewelt, Martin Dirksen-Fischer, Matthias Boldt, Volker Harth, Marcus Oldenburg

**Affiliations:** 1 Zentralinstitut für Arbeitsmedizin und Maritime Medizin (ZfAM), AG Schifffahrtsmedizin, Universitätsklinikum Hamburg-Eppendorf (UKE), Seewartenstraße 10 | Haus 1, 20459 Hamburg, Deutschland; 2 Hamburg Port Health Center des Instituts für Hygiene und Umwelt, Hamburg, Deutschland

**Keywords:** Arbeitsschutz, Pandemie, SARS-CoV‑2, Seeleute, Schifffahrt, Occupational safety, Pandemic, SARS-CoV‑2, Seafarers, Shipping

## Abstract

**Hintergrund:**

Eine Pandemie ist eine besondere medizinische Herausforderung für Seeleute, die ohne Arzt/Ärztin an Bord unterwegs sind. Gleichzeitig ist es eine Notwendigkeit für die weltweite Bekämpfung der COVID-19-Pandemie, Warenströme durch eine widerstandsfähige Handelsschifffahrt aufrechtzuerhalten. Für die Infektionsprävention und das Infektionsmanagement an Bord benötigen Verantwortliche ein Portfolio von Schutzmaßnahmen, die auf Schiffen angewendet werden können.

**Fragestellung:**

In der Übersicht wird der Fragestellung nachgegangen, welche technischen, organisatorischen und persönlichen Schutzmaßnahmen auf einem Handelsschiff angewandt werden können, um COVID-19-Ausbrüche an Bord zu verhindern oder bewältigen zu können.

**Material und Methoden:**

Richtlinien, Informationen und Arbeitsschutzstandards aus dem maritimen Setting, aber auch aus anderen Arbeitsbereichen wurden gesichtet, damit Verantwortliche diese angepasst an die Lage (z. B. Schiffsgröße, Ausstattung, Witterung, Betriebszustand, Arbeitsanforderungen, Kontakt mit Schiffsfremden, medizinische Probleme) variabel einsetzen können.

**Ergebnisse:**

Eine Handreichung, die konkrete, im maritimen Kontext erklärte technische, organisatorische und persönliche Schutzmaßnahmen für Crews zur anlassbezogenen Nutzung enthält, wurde erstellt. Kombinationsmöglichkeiten und Timing von Sicherheitsbarrieren werden darin zielgruppenorientiert erklärt.

**Fazit:**

Eine Fülle der aus arbeitsmedizinischer Literatur und den Erfahrungen des Hafenärztlichen Dienstes in Hamburg abgeleiteten Schutzmaßnahmen sind auf hoher See umsetzbar. Handelsschiffe sollten in Pandemiezeiten vorausschauend ausgestattet (z. B. mit Schnelltests) und Verantwortliche ermächtigt werden, begründete Infektionsschutzmaßnahmen angepasst an die Situation an Bord einzusetzen. Seeleute sollten unabhängig von ihrer nationalen Herkunft prioritäre Impfangebote erhalten.

## Abstract (en)

**Background:**

A pandemic is a particular medical challenge for seafarers traveling without a physician on board. At the same time the global fight against the coronavirus disease 2019 (COVID-19) pandemic has to rely on resilient merchant shipping in order to maintain the flow of goods. For infection prevention and management on board, those responsible need a portfolio of protective measures that can be used on ships.

**Research questions:**

The overview examines the question of which technical, organizational and personal protective measures can be used on a merchant ship in order to prevent or cope with COVID-19 outbreaks on board.

**Material and methods:**

Guidelines, information and occupational health and safety standards from the maritime setting, but also from many other work areas, were examined in order to facilitate a variable use by those responsible (e.g. according to ship size, equipment, weather, operating status, work requirements, contact with external persons, medical problems).

**Results:**

A handout was compiled that contains specific technical, organizational and personal protective measures for crews, explaining their use in a maritime context. Possible safety barriers, options to combine them and an appropriate timing are explained in a target group-oriented manner.

**Conclusion:**

A great variety of protective measures derived from occupational medical literature and the experience of the port medical service in Hamburg can also be implemented on the high seas. In pandemic times, merchant ships should be equipped with foresight (e.g. with rapid diagnostic tests) and those responsible should be authorized to use effective infection control measures adapted to the situation on board. Seafarers should receive priority vaccination offers regardless of their national origin.

Schiffsbesatzungen auf Handelsschiffen und ihre Arbeitgeber müssen sich dafür einsetzen, Infektionsrisiken zu minimieren. Auf See und in potenziellen Zielländern stehen oft nur eingeschränkte Behandlungsmöglichkeiten zur Verfügung. Seeleute leben und arbeiten an Bord über einen langen Zeitraum auf beschränktem Raum zusammen und haben in den Häfen nicht vermeidbaren Kontakt zu Schiffsfremden. Am Beispiel dieses Settings lässt sich darstellen, wie eine Organisationseinheit durch ein Portfolio von Schutzmaßnahmen befähigt werden kann, flexibel und adäquat auf sich verändernde Anforderungen durch die Pandemie zu reagieren.

## Hintergrund

### Sicherheitsbarrieren zur Vermeidung einer Infektion mit SARS-CoV-2

**Figure 1:**
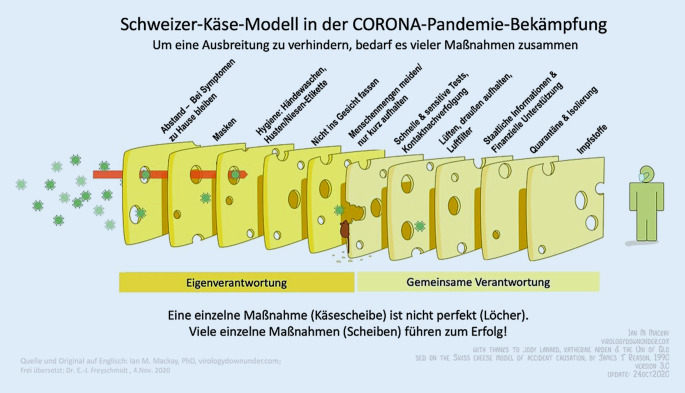

Das etablierte *Schweizer-Käse-Modell* für Unfallursachen von James Reason lässt sich auf das Infektionsgeschehen in der Pandemie mit dem Coronavirus SARS-CoV‑2 übertragen [28]. Die Erforschung der Übertragungswege von SARS-CoV‑2 erlaubt es, verschiedene Sicherheitsebenen zu benennen, die eines gemeinsam haben: Sie bieten bei alleinigem Einsatz keinen kompletten Schutz vor Infektion. Wie Löcher in den Scheiben eines Schweizer Käses weisen sie Lücken auf, die im ungünstigen Fall den Viruskontakt ermöglichen. Im günstigen Fall jedoch gleicht eine weitere Sicherheitsebene dieses Defizit aus. Möglichst viele Ebenen hintereinander erhöhen die Wahrscheinlichkeit der Widerstandsfähigkeit eines Systems und verhindern damit die Virusverbreitung (Abb. 1).

**Figure 2:**
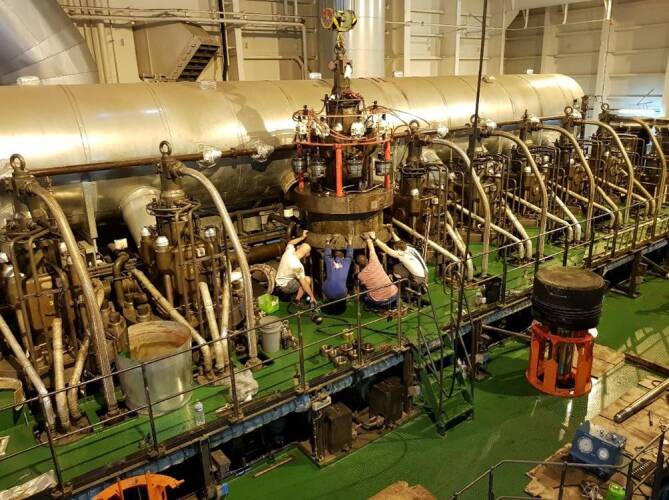

An Bord eines Frachtschiffes sind die gezeigten Sicherheitsebenen nicht in jedem Fall mit den notwendigen Arbeiten vereinbar. Bei einem Kolbenwechsel beispielsweise kann die Mannschaft die Abstandsregel bei den erforderlichen Arbeitsschritten nicht immer einhalten (Abb. 2).

Das heißt, unter Umständen können Maßnahmen, die zur Verminderung der Übertragungswahrscheinlichkeit sinnvoll wären (jederzeit Abstand halten, Quarantäne oder Isolation an Land), sogar die Schiffssicherheit gefährden (verschleppte Reparatur, zu geringe Besatzungsstärke).

Erwünschte Sicherheitsbarrieren sind nicht immer mit den notwendigen Arbeiten an Bord vereinbar

In dem Risikoanalysen und -management dienenden Modell von James Reason findet sich auch hierzu eine wichtige Information. Während die meisten Manager*innen traditioneller Systeme Fehler reflexhaft der menschlichen Unzuverlässigkeit zuschreiben und sich bemühen, Variabilität so weit wie möglich zu beseitigen, setzen sog. hochzuverlässige Organisationen (HRO – High Reliability Organisation) auf eine andere wichtige Sicherheitsvorkehrung: die Variabilität des Menschen in Form von Kompensationen und Anpassungen an sich ändernde Ereignisse [28].

### Herausforderungen durch die COVID-19-Pandemie und die besondere Situation an Bord

Eine speziell für das maritime Setting gefertigte Handreichung kann wertvolle Unterstützungsarbeit leisten, indem sie den Verantwortlichen an Bord (Kapitän*in oder Crewmitglied nach Delegation der Aufgabe), aber auch der Reederei, Betriebsärzt*innen und weiteren maritimen Stakeholdern mit Beratungsfunktion (u. a. Seemannsmissionen, Hafenagenturen, Terminalbetreibern) wissenschaftlich begründete und publizierte Handlungsoptionen aufzeigt, die Teil eines variablen und situationsadäquaten Schutzkonzeptes werden könnten. Es erfordert besonderes Fingerspitzengefühl der Verantwortlichen, Lösungen zu finden, die sowohl Sicherheitserfordernissen als auch sozialen Bedürfnissen an Bord gerecht werden. Auf einem Schiff fällt die Arbeits- und Lebenswelt über viele Monate hinweg zusammen. Eine Crew ist dennoch keine vollständig in sich geschlossene Gemeinschaft, denn durch unterschiedlich lange Vertragslaufzeiten kommen neue Crewmitglieder hinzu, und durch Hafenaufenthalte werden Außenkontakte, z. B. mit Mitarbeiter*innen von Hafenbehörden, Lotsendiensten, Lieferanten, erzwungen. Auch niedrige Inzidenzen an einem Zielort oder die fehlende Ausweisung als Risikogebiet erlauben es den Seeleuten bei dem dynamischen Geschehen nicht, von der Anwendung eines Schutzkonzeptes abzusehen. Die tatsächliche Inzidenz kann abhängig u. a. von Verfügbarkeit von Tests, zeitnaher Erfassung, öffentlicher Transparenz der Testergebnisse, aktueller Einschleppung von Mutanten stark von der tatsächlichen infektiologischen Lage und Gefährdung vor Ort abweichen.

### SARS-CoV-2-Infektionen im maritimen Setting

Fallbeschreibungen zeigen, dass SARS-CoV-2-Infektionen häufig symptomarm oder asymptomatisch verlaufen. So waren an Bord des Kreuzfahrtschiffes Diamond Princess im Frühjahr 2020 bei einem Ausbruchsgeschehen 44,8 % der positiv getesteten Crewmitglieder und 57,7 % der Passagiere asymptomatisch [26]. SARS-CoV-2-Infektionen auf Frachtschiffen werden in der wissenschaftlichen Literatur bisher eher selten untersucht [25]. Ein Ausbruch auf einem Containerschiff im Februar/März 2020 zeigt, dass eine Crew mehr als 14 Tage benötigen kann (in Deutschland derzeit empfohlene Zeitspanne für eine häusliche Absonderung – „Quarantäne“; [29]), bis Zeichen einer SARS-CoV-2-Infektion unter Crewmitgliedern wahrgenommen werden (Tag 22 nach Boarding) oder der Infektionsnachweis gelingt (Tag 27 nach Boarding, [7, 35]). Erschwerend kommt hinzu, dass eine labortechnische Verifizierung einer SARS-CoV-2-Infektion mittels einer RT-PCR („reverse transcription polymerase chain reaction“) nach einem korrekt durchgeführten Nasen-Rachen-Abstrich als Goldstandard in der Diagnostik [13] an Bord nicht durchführbar ist. Seeleute, die auf Handelsschiffen für gewöhnlich ohne Ärztin/Arzt an Bord unterwegs sind, können bei medizinischen Fragen zu dem Umgang mit der Erkrankung, persönlicher Schutzausrüstung (PSA) und Testmöglichkeiten Unterstützung u. a. bei einem weltweiten Netz von funkärztlichen Beratungsstellen (TMAS – Telemedical Maritime Assistance Service) in Anspruch nehmen.

In einer Crew können weit mehr als 14 Tage vergehen, bis Infektionszeichen wahrgenommen werden

Selbst die von vielen maritimen Stakeholdern geforderte [20, 23], nationalitätenübergreifende [34] und systematische Impfung von Seeleuten [19], stellt lediglich eine der Sicherheitsebenen des adaptierten James-Reason-Modells dar (Abb. 1). Ihr Effekt ist u. a. abhängig von der Wirksamkeit des jeweiligen Impfstoffes gegen das Ursprungsvirus oder eine Mutante, dem Impfprozess, der Reaktion des Individuums und der Immunität der Gesamtbevölkerung.

## Methoden

Die hochdynamische Verbreitung von SARS-CoV‑2 erfordert eine besondere Beachtung spezifischer Richtlinien, welche aufgrund der Aktualität laufend auch für den maritimen Kontext überprüft werden müssen. Aktuelle Informationen über Fragen zum Umgang mit SARS-CoV‑2 sind auf der Website der WHO (World Health Organisation) sowie des RKI (Robert Koch-Institut) oder des ECDC (European Centre for Disease Prevention and Control) verfügbar und stellen eine Grundlage der in der Anmerkung verlinkten Handreichung und dieses Textes dar. Über die Schutzmaßnahmen an Bord von Handelsschiffen gibt es derzeit wenig gesicherte wissenschaftliche Evidenz, so dass viele der vorliegenden Empfehlungen [18, 36] auf den aktuellen Ausführungen der genannten Institutionen sowie der EU-Forschungsgruppe Healthy GateWays und dem SARS-CoV-2-Arbeitsschutzstandard bzw. der Arbeitsschutzregel des Bundesministeriums für Arbeit und Soziales (BMAS) beruhen. Durch viele Hyperlinks zu diesen Quellen und zu weiteren etablierten Informationsmaterialien für die Crew erleichtert eine Handreichung den Verantwortlichen die praktische Nutzung auf hoher See.

## Portfolio technischer, organisatorischer und persönlicher Schutzmaßnahmen

### Technische Schutzmaßnahmen

#### Raumlufttechnische Anlagen

Der Wohnbereich auf Handelsschiffen wird durch raumlufttechnische Anlagen (RLT) belüftet und ggf. klimatisiert. Diese Anlagen sollten an Bord ausschließlich mit Außenluft und nicht mit Umluft betrieben werden. Insbesondere bei Räumen, in denen Infizierte behandelt oder isoliert werden (z. B. im Bordhospital von Handelsschiffen), muss ein Umluftbetrieb der RLT vermieden werden, da hierdurch die Aerosolkonzentration in der Raumluft erhöht wird. Unter Frischluftzufuhr kann das Infektionsrisiko deutlich gesenkt werden [4, 10]. Die Handlungsanleitung zur Hygiene und Wartung von RLT auf Seeschiffen birgt weitere Informationen als Hilfestellung bei der Wartung [17].

#### Abtrennungen aus transparentem Material

Kann die Abstandsregel zwischen Arbeitsplätzen aus betriebstechnischen Gründen nicht eingehalten werden, ist als technische Maßnahme die Installation von Abtrennungen zu prüfen [3]. Durch die Abtrennungen darf es nicht zu zusätzlichen Gefährdungen kommen. Ihre Installation ist generell, aber insbesondere für Innenräume zu prüfen, in denen es zu einem kommunikativen Austausch mit Schiffsexternen kommt [36].

#### Wirksame Desinfektionsmittel gegen SARS-CoV-2

Kontakte erfordern die Notwendigkeit zum regelmäßigen Händewaschen mit Wasser und Seife, alternativ die Vorhaltung von Händedesinfektionsmitteln, mindestens mit dem Standard „begrenzt viruzid“ [14]. Eine anlassbezogene (Arbeitsplatz von Schiffsexternen) und regelmäßige Flächenreinigung ist insbesondere auch bei gemeinsam genutzten Oberflächen (Handläufe, Türgriffe, Kartentisch, Cockpit-Armaturen), in der Schiffskombüse und in den Sanitäranlagen sinnvoll [14]. Durch Reinigungs- und Hygienepläne soll die Frequenz und die Zuständigkeit dokumentiert werden.

Zur Desinfektion von medizinisch genutzten Bereichen an Bord (z. B. im Schiffshospital) ist eine Liste wirksamer Viruzide gegen unterschiedliche Coronaviren den Leitlinien des ECDC, des RKI und der US-Umweltschutzbehörde (EPA) zu entnehmen [30].

### Organisatorische Schutzmaßnahmen

An Bord von Handelsschiffen kommt der Umsetzung organisatorischer Schutzmaßnahmen zur Infektionsverhütung und zur Infektionskettenunterbrechung eine große Bedeutung zu (z. B. auch AHA + L-Regel ≙ Abstand, Hygiene, Atemschutz und Lüften). Da Arbeits- und Lebensort an Bord zusammenfallen, müssen Arbeitsschutzregeln u. U. an die jeweilige Situation angepasst werden.

#### Schutzkonzept und Hygieneprotokollführer*in bei Kontakt mit externen Personen

Während der Pandemie sollte ein Schutzkonzept für jedes Schiff festgelegt und die Umsetzung der notwendigen Schutzmaßnahmen durch ein*e Hygieneprotokollführer*in dokumentiert werden (z. B. Kontaktdaten, Kontaktpersonen an Bord, Zeitraum der Anwesenheit und angewandte Hygieneregeln).

Kontakt zu schiffsexternen Personen sollte soweit möglich reduziert werden und ggf. unter Einhaltung der AHA + L-Regeln stattfinden. Erfordern die Umstände doch die gemeinsame Nutzung von Innenräumen, müssen diese, wenn möglich, durch Stoßlüftung gewissenhaft ventiliert werden. Übergaben sollten, wann immer möglich, im Freien, schriftlich oder telefonisch erfolgen.

#### Arbeitszeit- und Pausengestaltung

Zur Verringerung der Personenkontakte auf einem Schiff soll die Nutzung gemeinsamer Arbeitsplätze oder Räume, wie z. B. Messe, Fitnessraum, Gemeinschaftsduschen oder Umkleideräume zeitlich getrennt erfolgen und möglichst dieselben Besatzungsmitglieder in gemeinsamen Arbeits- bzw. Schicht-Kohorten organisiert werden.

Übergaben sollten, wann immer möglich, im Freien, schriftlich oder telefonisch erfolgen

Informationsaustausch z. B. im Rahmen einer Übergabe sollte, wann immer möglich, im Freien, schriftlich oder telefonisch erfolgen. Ist der Aufenthalt im Freien problematisch, da eine Infektionsgefahr durch Insektenstiche besteht, können es Mosquitoscreens erlauben, von dem eigentlich empfohlenen Schließen der Fenster und Türen abzusehen.

#### Einhaltung ausreichender Schutzabstände

Alle sicheren Fußwege sollten benutzt und frequentierte Wege (z. B. Treppen) sollten so begangen werden, dass ein ausreichender Abstand zwischen dem Crewpersonal eingehalten werden kann. Daher ist nach Möglichkeit auch auf die Verwendung von Aufzügen zur gleichzeitigen Beförderung mehrerer Personen zu verzichten [6]. An Orten, an denen mehrere Personen zusammentreffen können (z. B. an der Gangway), sollen Schutzabstände der Stehflächen z. B. mit Klebeband gekennzeichnet werden. Der Mindestabstand von 1,5 m soll bei der Zusammenarbeit mehrerer Besatzungsmitglieder, wie z. B. bei Anlegemanövern, und auch in der Freizeit eingehalten werden. Wenn dieses nicht möglich ist, ist eine Schutzmaske, idealerweise (K)N95- oder FFP2-Maske, zu tragen [3, 4, 32].

#### Crewwechsel

Bei neu anmusternden Besatzungsmitgliedern darf kein Verdachtsfall einer COVID-19-Erkrankung bestehen. Hierfür sollten Selbstquarantäne im Heimatland, Vorsichtsmaßnahmen bei der Anreise, Selbstauskunft vor Boarding [36] und PCR-Tests auf eine vorliegende Infektion mit qualifizierter Probenentnahme und -untersuchung als weitere Mittel der Risikominimierung eingesetzt werden [21]. Verlängern sich Laufzeiten der Kontrakte überraschend, sollte von Seiten der Reederei alles dafür getan werden, um psychischen Stress für die an Bord verharrenden Seeleuten zu reduzieren (z. B. durch offene Kommunikationskanäle zur Kontakterhaltung zu Familie und Freunden, Ermöglichung der Kontaktaufnahme zur lokalen Seemannsmission, aber auch ggf. Zugang zu benötigter Dauermedikation [18]).

#### Arbeitsmittel/Werkzeuge

Werkzeuge und Arbeitsmittel sollten personenbezogen verwendet werden. Kann dies nicht gewährleistet werden, ist eine regelmäßige Reinigung vor der Übergabe und Händewaschen nach Nutzung sinnvoll (s. auch Abschnitt „Wirksame Desinfektionsmittel gegen SARS-CoV-2“). Andernfalls sind bei der Verwendung der Werkzeuge geeignete Schutzhandschuhe zu verwenden, sofern hierdurch nicht zusätzliche Gefahren (z. B. Erfassung durch rotierende Teile) entstehen [4].

#### Schnelltests auf Antigenbestandteile von SARS CoV‑2

Zur Unterbrechung von Infektionsketten und Verbesserung des Krankheitsmanagements sollten Schnelltests (Antigen Detection Rapid Diagnostic Tests, Ag-RDT) in ausreichender, an der Besatzungsstärke orientierter Menge an Bord vorgehalten werden. Positive Schnelltests können innerhalb eines Zeitraums von 10–30 min auf eine Infektion mit SARS-CoV‑2 hinweisen [13, 15].

Mögliche Vorteile des Einsatzes von Schnelltests an Bord sind z. B.:

- Schnelltest sprechen in der Latenz- und frühen Infektionsphase auf Grund der dann gewöhnlich hohen Viruslast gut an und ermöglichen so den frühzeitigen Nachweis einer Infektion (Zeitraum 1–3 Tage vor dem Auftreten von Symptomen und in der frühsymptomatischen Phase innerhalb der ersten 5–7 Tage der Krankheit [11, 13]).
- Ein positives Ergebnis muss in die schiffsseitig auszufüllende Maritime Declaration of Health (MDH, Seegesundheitserklärung) eingetragen werden. Sie dient der rechtzeitigen Information des nächsten Hafens über gesundheits- oder hygienebezogene Besonderheiten an Bord. Aus Perspektive der Crew und der Reederei besteht der Vorteil darin, dass Zeit eingespart und eine prompte und adäquate medizinische Reaktion und ggf. klinische Versorgung nach Ankunft im Hafen möglich wird (Abstrich nach Anlegemanöver und Durchführung und Analyse eines PCR-Tests im Labor).
- Ag-RDTs vor dem Einlaufen in den Hafen (auch von Kontaktpersonen an Bord) erleichtern und verbessern Entscheidungen aller Funktionsträger im Fallmanagement.
- Schnelltests können vor Kontakten mit Lotsen, Hafenbehörden und anderen Schiffsexternen angewandt werden und so die Schiffsabfertigung sicherer machen.
- Bietet ein Hafen keine schnellen RT-PCR-Tests, die sensitiver sind als Ag-RDT, hat ein mit Schnelltests ausgestattetes Schiff eine diagnostische Alternative zur Verfügung.
- Besteht kein Zugang zu Laboruntersuchungen auf Virus-RNA mittels RT-PCR, können Schnelltests auch für Infektionsverläufe an Bord (z. B. Umschlag eines positiven in ein negatives Ergebnis) eingesetzt werden [15].

Es wird empfohlen, nur die vom Paul-Ehrlich-Institut (PEI) gelisteten Schnelltests zu verwenden [27] und zur Beurteilung der Ergebnisse sowie zur Planung des weiteren Vorgehens eine Beratung durch die für Gesundheitsfragen zuständigen Hafenbehörden oder TMAS, z. B. den Funkärztlichen Beratungsdienst Cuxhaven, hinzuzuziehen.

#### Definition und Handlungsanweisungen für Verdachtsfälle und enge Kontaktpersonen

Wenn eine Person an Bord, die in der Handreichung beschriebenen und in den aktuellen Klassifikationen des RKI und ECDC festgehaltenen Kriterien erfüllt, sollte sie sofort in der eigenen Kammer isoliert, ein Schnelltest auf Virusantigen durchgeführt, Kontaktpersonen identifiziert und solche mit engem Kontakt („high risk exposure“) quarantänisiert werden [8]. Das International Chamber of Shipping klassifiziert zumindest die folgenden Personen als Verdachtsfälle [18]:

- Nutzer derselben Kabine,
- Crewmitglieder, die sich einen Meter nahe gekommen sind oder in einem geschlossenen Umfeld waren (z. B. gemeinsam gegessen haben, zusammen am Tank arbeiteten oder in einem Maschinen-Kontrollraum Wache hatten),
- Crewmitglieder, die gemeinsam angereist sind,
- das Crewmitglied, das die Kabine eines Verdachtsfalls gereinigt hat und
- die medizinischen Betreuenden des Verdachtsfalles.

Die zuständigen Behörden des nächsten Anlaufhafens müssen informiert werden [16] und entscheiden, ob die gesamte Besatzung unter Quarantäne gestellt werden muss. In diesem Zuge sollte auch in Erfahrung gebracht werden, ob die erforderlichen Kapazitäten (z. B. für Abstrich an Bord, möglicher Ausstieg, Transport, Isolierung und Betreuung) im Hafen verfügbar sind [16]. Es muss auch bei negativem Schnelltest ein landseitiger RT-PCR-Test zur Validierung des Ergebnisses angestrebt werden. Während der Influenzasaison ist ein ergänzender Test auf das Grippevirus sinnvoll [11]. Ein kleiner Kreis von Betreuenden sollte festgelegt und verpflichtet werden, immer persönlich Schutzausrüstung (PSA) zu tragen, wenn sie Kontakt mit den Betroffen haben und diesen zu dokumentieren [12, 16]. Eine Dekontaminierungszone sollte eingerichtet werden [9]. Telefonische oder Chat-Austauschmöglichkeiten mit isolierten Besetzungsmitgliedern sollten ebenfalls genutzt werden. Sind ausreichend Ag-Schnelltests an Bord vorhanden, sollte die gesamte Crew untersucht und die Testung ggf. nach einigen Tagen, bei Auftreten von Symptomen sofort, wiederholt werden. Wenn eine erkrankte und/oder eine als enge Kontaktperson identifizierte Person das Schiff verlassen muss, ist auch in dieser Phase jeglicher Kontakt mit anderen Besatzungsmitgliedern oder anderen Personen auf ein Minimum zu reduzieren und PSA zu tragen. Wäsche, Küchenutensilien und Abfälle aus den Kammern von Verdachtsfällen und Kontaktpersonen [8] sollen als infektiöses Material behandelt werden [16].

#### Psychische Belastungen minimieren

In der Schifffahrt entstehen derzeit besondere Herausforderungen durch die aufgrund von COVID-19 resultierenden Reiseeinschränkungen: Seeleute müssen über ihren mehrmonatigen Dienst hinaus ihren Aufenthalt an Bord verlängern, da sie entweder nicht ersetzt werden oder nicht in ihre Heimat zurückfliegen können [22]. Diese Rahmenbedingungen wirken sich nicht nur auf sicherheitsrelevante Aspekte an Bord, sondern auch auf die psychische Konstitution und das Wohlergehen der Seeleute aus [33]. Sorgen und Ängste müssen wahrgenommen und auf individuelle Bedürfnisse eingegangen werden [24].

Der Aufenthalt an Bord wird auf Grund der COVID-19-Pandemie oft auf unbestimmte Zeit verlängert

An Bord herrschen viele weitere psychische Belastungsfaktoren vor, deshalb enthält die Handreichung Verweise auf Angebote in Krisen (z. B. durch Seemannsmissionen, das International Seafarers’ Welfare & Assistance Network [ISWAN], oder Seafarer Crisis Action Team [SCAT] der International Maritime Organisation [IMO]). Eine landbasierte Quarantäne kann die psychosoziale Betreuung der Seeleute und die Kooperation mit den benannten Institutionen erleichtern [35].

### Persönliche Schutzausrüstung (PSA)

An Bord sollten über die bestehenden Bestände medizinischer Ausrüstung gemäß nationaler Vorgaben hinaus ausreichende Vorräte an personenbezogen genutzter PSA vorgehalten werden, dazu zählen [4, 5, 16]:

- Einweghandschuhe,
- langärmlige und undurchlässige Schutzkleidung,
- Schutzbrillen bzw. Gesichtsschutz (Schutzschilde, -visiere),
- Mund-Nasen-Bedeckung,
- FFP2/FFP3-Masken (oder (K)N95).

Wenn Abstände nicht sicher eingehalten werden können (z. B. bei Team-Arbeitsprozessen im Maschinenraum oder auf der Brücke), sollen Mund-Nasen-Bedeckungen zur Verfügung gestellt und getragen werden. Durch FFP2-Masken mit Ausatemventil können virushaltige Aerosole weitgehend ungefiltert freigesetzt werden. Sie stellen somit unter Umständen eine Fremdgefährdung dar. Masken ohne Ventil dagegen filtern sowohl die eingeatmete Luft als auch die Ausatemluft und bieten daher sowohl einen Eigenschutz als auch einen Fremdschutz [2]. Bei der Verwendung von PSA durch Lotsen und anderen Personengruppen im Bereich der maritimen Wirtschaft sind Fremdschutz und Eigenschutz sowie die entsprechenden arbeitsmedizinischen und weitere Vorgaben entsprechend zu berücksichtigen.

Masken mit Ventil filtern nur die eingeatmete Luft und sind nicht für den Fremdschutz ausgelegt

Handschuhe, Schutzkleidung, Gesichtsschutz und FFP2/FFP3-Masken sollen darüber hinaus getragen werden bei [4, 16]:

- dem Umgang mit Personen mit Infektionsverdacht,
- der Durchführung eines Schnelltests [1],
- Reinigung von Räumen, in denen sich Infizierte bzw. Personen mit Infektionsverdacht aufgehalten haben,
- dem Umgang mit potenziell infektiösen Abfall (dazu zählt auch getragene Einweg-PSA und Filter von RLT).

Das an einer Kontaminationswahrscheinlichkeit orientierte 4‑Zonen-Modell der WHO kann die Beratung zum Einsatz von Schutzausrüstung auf Frachtschiffen unterstützen [36]. Für den Fall, dass es zu Engpässen an Bord kommen sollte, erleichtern Empfehlungen des Robert Koch-Institutes zum ressourcenschonenden Einsatz von Masken im Gesundheitssektor und Entscheidungen bezüglich deren Wiederverwendung [31].

## Diskussion

In der aktuellen COVID-19-Pandemie diktieren inzwischen bekannte Eigenschaften des SARS-CoV-2-Ursprungsvirus und der bisher aufgetretenen Mutationen neue arbeitsmedizinische Empfehlungen. Nur wenn sehr ähnliche Viruseigenschaften vorherrschten, wären die aktuellen Erkenntnisse auch auf andere Infektionsgeschehen unverändert übertragbar. Empfehlungen für das maritime Setting müssen während einer Pandemie nicht nur auf Virusbesonderheiten maßgeschneidert und in ihrer Wirkung verständlich, sondern sowohl für die Prävention, als auch – nach deren Teilversagen – für das Management eines Ausbruchs an Bord geeignet sein. Eine umfassende Orientierungshilfe ermöglicht es, trotz variierender Voraussetzungen, angemessen zu reagieren und das Vorgehen durch Verweis auf anerkannte Handlungsempfehlungen zu rechtfertigen. Sie kann Verantwortliche an Bord auch dabei unterstützen, Handlungsalternativen abwägen, um den erforderlichen Ausgleich zwischen Sicherheitsbestreben und sozialen Bedürfnissen herzustellen. Das Wissen um diese vielfältigen Anwendungsszenarien für arbeitsmedizinische Empfehlungen und Nichtkennen der besonderen Herausforderungen für die Schiffssicherheit in der jeweiligen Situation erlauben es nicht, über das Aufzeigen eines Portfolios von Möglichkeiten hinauszugehen.

Dieses Vorgehen kann für Arbeitsmediziner*innen, die ähnlich exponierte, intermittierend isolierte und/oder selbstverantwortliche Settings betreuen, interessant und beispielhaft zitierbar sein. Eine der arbeitsmedizinischen Logik folgende Darstellung (technische, organisatorische und persönliche Schutzmaßnahmen bzw. Ausrüstung) lädt zur fachlichen Auseinandersetzung und erfahrungsbasierten Weiterentwicklung ein und ist so dazu geeignet, professionell Handlungssicherheit bei für den Arbeitsschutz verantwortlichen Personen in besonderen Settings aufzubauen. Einen arbeitsmedizinisch begründeten Handlungskorridor aufzuzeigen, ist insbesondere immer dann wichtig, wenn Arbeitsschutzstandards oder Erkenntnisse aus Studien zum Management von COVID-19-Ausbrüchen nicht vollumfänglich auf die spezifische Arbeitssituation übertragbar sind.

## Fazit und Empfehlungen

- Die COVID-19-Pandemie stellt eine gesundheitliche Notlage von internationaler Tragweite dar. Die Handelsschifffahrt ist zentral davon getroffen.
- Die beschriebenen technischen, organisatorischen und persönlichen Schutzmaßnahmen und Empfehlungen können die Ausbreitung der Pandemie und die Auswirkungen auf den Schiffsbetrieb reduzieren.
- Sie sollten von den Verantwortlichen (Kapitän*in oder Crewmitglied nach Delegation der Aufgabe) nach ihrer Implementierung auf ihre Wirksamkeit überprüft werden. Maritime Ausrüstungsverzeichnisse müssen angepasst werden, wenn eine Maßnahme erfolgreich ist (z. B. Mitführen von Schnelltests).
- Die dynamische Entwicklung und Verbreitung von SARS-CoV‑2 erfordert wiederholte Risikoeinschätzungen durch die Verantwortlichen sowie eine ständige evaluationsbasierte Weiterentwicklung und Anpassung dieser Maßnahmen durch Experten (inklusive der Empfehlung günstiger schiffsbaulicher Voraussetzungen).
- Ein wichtiger, nächster Meilenstein bei der Bekämpfung der Auswirkungen von Pandemien auf die Handelsschifffahrt und Warenströme ist die breite Impfung auch durch Arbeitsmediziner*innen, derzeit insbesondere die Applikation eines Einmalimpfstoffes gegen SARS-CoV‑2, aber auch von jährlichen Influenzaimpfungen bei Seeleuten, unabhängig von ihrer nationalen Herkunft.
